# The Role of Insect-Based Feed in Mitigating Climate Change: Sustainable Solutions for Ruminant Farming

**DOI:** 10.3390/insects16050516

**Published:** 2025-05-13

**Authors:** Nelly Kichamu, Putri Kusuma Astuti, Szilvia Kusza

**Affiliations:** 1 Centre for Agricultural Genomics and Biotechnology, University of Debrecen, 4032 Debrecen, Hungary; kichamu.nelly@agr.unideb.hu (N.K.); astuti@agr.uninedb.hu (P.K.A.); 2 Doctoral School of Animal Science, University of Debrecen, 4032 Debrecen, Hungary; 3 Ministry of Agriculture Livestock, Fisheries and Cooperatives, Directorate of Livestock Development, Naivasha Sheep and Goats Breeding Station, Naivasha P.O. Box 2238-20117, Kenya; 4 Department of Animal Breeding and Reproduction, Faculty of Animal Science, Universitas Gadjah Mada, Yogyakarta 55281, Indonesia

**Keywords:** alternative feeds, insects, climate change mitigation, sustainable livestock farming

## Abstract

There has been an unprecedented demand for livestock production due to factors such as the ever-increasing population, limited resources (land, water, feed, etc.), and changing human lifestyles. Moreover, due to the interconnected nature of the world’s biodiversity crisis, pollution, and climate change, environmental sustainability is going to play a pivotal role in addressing these pressing issues. Because of their high nutritional value and environmental benefits compared to conventional livestock feeds, insects as animal feed have demonstrated great potential for long-term sustainability. The current state of the IBF application on ruminants is presented in this review, together with its challenges, future direction, and strength–weakness–opportunity–threat analysis. The results from many studies on ruminants have demonstrated that insect nutrients—primarily amino acids, protein, and fat—are highly digestible, safe, and beneficial to ruminant health and productivity. Additionally, they do not harm the ruminant fermentation and microbiota, even having the benefit of possibly lowering ruminant farms’ well-known methane emissions. Nevertheless, concerns continue to arise because this method is still relatively new and there is a lot of unexplored knowledge; as a result, regulation is not yet well established globally, which is a barrier to its implementation.

## Simple Summary

Insects have gained popularity as a livestock feed alternative to conventional feeds in the past years owing to their high nutritional value and environmental benefits. Not only are insects a great source of protein, but their production also leaves a significantly smaller carbon footprint and requires fewer resources (land, feed, water, transportation fuel, and human labour) than that of conventional feed sources, making them a sustainable solution. Nevertheless, in order to increase the adoption of this sustainable feeding practice, it is necessary to establish regulations for the farming and cultivation of insects, and their application as fodder for livestock. With such effective regulation, consumer acceptance of livestock products fed with insects would improve, potentially encouraging farmers to adopt this practice.

## 1. Introduction

When we talk about sustainable livestock feeding, we are referring to methods that try to keep the animals healthy while minimizing the negative effects on the environment. Nutritional composition, antinutritional elements, digestibility, and palatability are the determining factors in feed resources. Additionally, the economic viability of feed formulations must be considered, including the costs of the ingredients involved in their preparation, as this directly impacts the feasibility of sustainable practices, which, in turn, affect livestock health, performance, and overall production efficiency. Feed represents the most critical and significant factor in livestock farming operational cost (65–80% of total cost) and must be precisely adjusted to meet the animals’ energy needs, preventing overfeeding and the resultant nutrient waste in the environment [1,2].

The production of methane by livestock, particularly ruminants, has been the subject of much debate and discussion for quite some time, including in relation to its mitigation through nutrition management. Research on alternative feeding resources for the reduction in methane emissions in ruminants has been extensive; some studies have proposed aquatic weeds [3], algae [4], and various plant extracts as feed additives [5,6,7]. Several challenges, including high production costs, sustainability concerns, and lack of availability, hinder the widespread use of these alternate feeds.

Insects are a kind of arthropod with a chitin exoskeleton and a body composed of three segments: the head, the thorax, and the abdomen. Despite being one of the most undervalued feed resources and mainly being included in traditional delicacies, especially in the tropics [8,9,10], these groups of organisms are among the most diversified [11]. Pollination, composting, protecting against wildfires, controlling pests, and providing food for animals and humans are just a few of the important ecosystem services they provide. An increasing amount of research suggests that insects could provide a sustainable alternative to animal protein (40–60%) and fat (30–40%) sources to replace the current overreliance on fishmeal (FM) and soybean meal (SBM), with their high feed conversion efficiency being a key argument in their favor [12,13].

When it comes to solving many problems with modern farming, including the issues of methane emission and sustainability, insects provide a very sustainable solution; their ability to convert organic waste materials like food waste, agricultural by-products, and manure into biomass that is high in protein is advantageous. Insects are a more eco-friendly alternative to conventional feed sources for livestock as their use leads to more efficient water usage and less land degradation. Insect farming can generate protein at much reduced environmental costs and thus the cost of production is reduced. Livestock farmers can reduce their operations’ negative effects on the environment without sacrificing the nutritional balance their animals need to thrive by using insects in their feed [14,15]. This method can aid in the establishment of a food system that is more resilient and sustainable, which, in turn, can assist in reducing the strain on conventional feed sources and advance global sustainability objectives.

This review aims to summarize the most recent research on insect-based feed (IBF) for various livestock species with an emphasis on how it can help in methane emission reduction. The influence of IBF on the environmental footprint of the livestock sector is examined in this review, as well as the role of IBF in reducing the impact of climate change on livestock, which is more resource-efficient and beneficial to the environment. To a lesser extent, IBF aids larger initiatives to lessen the environmental impact, water consumption, and land degradation linked to traditional feed crops by advocating for more environmentally friendly feed production practices. We compare conventional feed sources with IBF, its nutritional benefits and varied uses in ruminant farming, and its implementation for various production methods, including some of the drawbacks and challenges that come with using IBF, followed by an evaluation suggesting opportunities for further study and development. The results obtained in this review are presented below.

## 2. Materials and Methods

The conventional literature review methodology was used for this research due to its adaptability and exploratory qualities. The following terms/keywords were used in various different combination aiming to find relevant information: “ruminants”, “cattle”, “sheep”, “goats”, “mealworms”, “BFSL”, “crickets”, “grasshoppers”, “houseflies”, “methane emission”, “insect feed”, and “insect-based feeding”. Web of Science (WoS), Scopus and Google Scholar were used. No specific timeframe restriction was employed in filtering the relevant publication. Research articles from reputable scholarly journals, both in as reviews and original research, were given priority in the literature selection process. Publications that did not have full-text accessibility or were not published in English were not excluded. Data visualization was executed using Power Point (Microsoft Cooperation, Redmond, WA, USA).

## 3. Effect of Conventional Protein-Based Livestock Feed on the Environment

**Figure 1:**
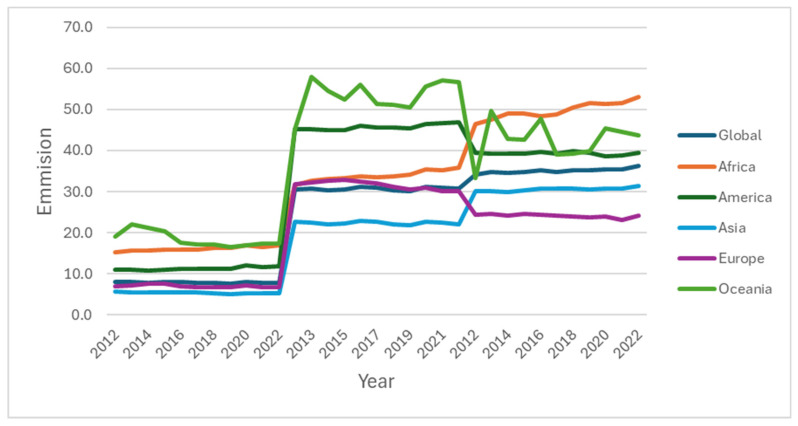
Total greenhouse gas emissions attributed to livestock production by regions in million metric tonnes of carbon dioxide equivalent (MtCO_2_e) [23].

Conventional protein-based livestock feeds are feeds given to livestock and are composed of a mixture of ingredients designed to meet the dietary needs of these animals. The feeds often include human-edible components, which bring about competition with humans in terms of consumption; examples of these feeds include sunflower cake, FM, and SBM, among others [16]. The production and usage of conventional animal feed has caused environmental issues: pollution, climate fluctuation, and depletion of natural resources [17]. This is brought about by the amount of manure that is produced by the animals fed on these conventional feeds. Generally, livestock are categorized as one of the highest contributors to greenhouse gas emissions [18]; as Figure 1 shows, in the emission trends for 11 years, between 2012 and 2022, for different regions of the world, all the regions showed an increasing trend in greenhouse gas emissions, particularly in Africa and Asia.

Cattle are the highest producers of methane through enteric fermentation during the digestion process, and through their manure, which releases both methane and nitrous oxide to the environment [19], which has a higher global warming impact, accounting for 296 times more emissions from carbon dioxide, and it remains in the atmosphere for up to 150 years. In addition, the global warming potential from methane is said to be 23 times higher as compared to that from carbon dioxide. From the studies, 4.5% of the total global GHG emission is from livestock production [20]. Furthermore, the production of these conventional livestock protein feeds, like soya, sunflowers, etc., needs a vast amount of land, which needs to be created through deforestation, burning of the bushes and vegetation-clearing to create space for production [21]. Additionally, for this feed to grow well, it needs fertilization (mostly inorganic fertilizer and herbicides), which, in turn, affects the environment through residues. In addition, the excess use of fertilizers may drain into water bodies, thus affecting the marine ecosystem. Animal operations also degrade air quality by emitting pollutants such as ammonia (NH_3_), hydrogen sulfide (H_2_S), and particulate matter (PM_2.5_) from manure management and enteric fermentation. These emissions are linked to respiratory diseases and environmental harm. The connection to conventional protein-based feeds (e.g., soy, corn) lies in their resource-intensive production: synthetic fertilizers release nitrogen oxides (NO_x_), while land use changes for feed crops contribute to biomass burning emissions. Insect-based feeds offer a sustainable alternative by reducing reliance on such inputs, thereby mitigating air pollution across the livestock production chain [22].

## 4. Insects as an Alternative Sustainable Feed Resource

**Figure 2:**
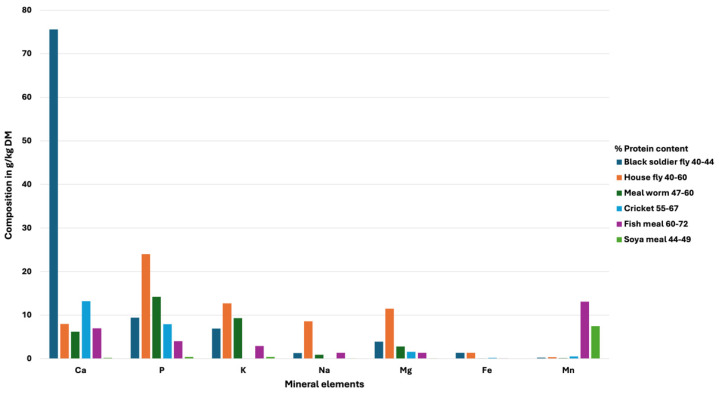
Comparison between the mineral composition of insect protein and conventional protein meals. Calcium (Ca); phosphate (P); potassium (K); sodium (Na); magnesium (Mg); iron (Fe); manganese (Mn) [31].

Because of their rapid development and remarkable efficiency, insect farming for feed is a more sustainable alternative to conventional feed production [24]. When compared to animals, insects are far better at breaking down organic materials into protein; thus, farming insects can help alleviate the issue of food loss and waste. With the growing demand for sustainable animal production, insect-based feed (IBF) has emerged as a climate-smart alternative to conventional protein sources. By replacing resource-intensive feeds like soybean meal (SBM) and fishmeal (FM), IBF significantly reduces greenhouse gas emissions, land use, and the water footprint associated with livestock farming [25]. Besides environmental benefits, IBF offers a superior nutritional value, including high protein (40–70% dry weight) with complete amino acids levels, beneficial lipids such as omega-3/6, and important micronutrients such as iron, calcium, potassium, sodium and magnesium and prebiotic chitin. Black soldier fly larvae provide high calcium for bone health, housefly maggots are rich in phosphorus for energy metabolism, and mealworms/crickets supply potassium for cellular functions [26,27,28] (Figure 2). These dual advantages, mitigating climate change while enhancing feed efficiency, position IBF as an important solution for sustainable ruminant production.

Insect-based proteins not only reduce greenhouse gas emissions compared to conventional feeds (Table 1), but also contain chitin, a bioactive compound that enhances livestock immunity by lowering antibiotic use in ruminant production; chitin-rich insect feed contributes to mitigating antimicrobial resistance (AMR), which is a critical driver of environmental degradation in livestock systems [29,30]. Thus, the dual benefits of emission reduction and improved herd health position insect-based feed as a holistic climate-smart solution.

**Table 1:** Comparative GHG emissions (IBF vs. conventional feeds).

Feed Type	kg CO_2_-Equivalent	References
General insect	−6.42 to 5.3 (food waste) 0.77–12 (manure)	[32]
Mealworms	1.47	[33]
House crickets	110	[33]
Fishmeal	2–4	[24,33,34]
Soymeal	0.65–1	[33,34,35]
Fish meal	2–4	[32]
Black soldier fly	0.91	[34]
Cricket	1.3–2.9	[36]

## 5. Common Types of Insects Used as Livestock Proteins

Several insect species are being recognized for their potential as animal feed in terms of resource use, emissions, and waste conversion efficiency (Table 2); an example of this includes the Black soldier fly (*Hermetia illucens*) (BSF), which is categorized as a Diptera of the family Stratiomyidae. Though originally native to the Americas, it is now found worldwide in tropical and temperate regions [37]. An adult fly is black and looks like a wasp with a length of 15–20 mm long, The larvae measure approximately 27 mm in length and 6 mm in width and can weigh up to 220 mg at their last larval stage, in which they present themselves as dull, whitish in color, and mature within two months [38]. The flies do not feed on anything apart from water and are mild, and hence not harmful to humans [39]. Black soldier fly larvae (BSFL) feed on organic wastes such as manure substrates, kitchen wastes and rice straw [40,41]. Males stay at the lekking sites, where they meet the flying females. After females are mated, they only take two days to lay eggs in the prepared organic waste and die [42]. BSF has been mostly used as poultry, fish, pigs, and pet food as live, chopped, or dried and ground.

Mealworms (*Tenebrio molitor*) belong to the family Tenebrionidae, and are also known as yellow worms, which is a larval form of common grain beetle [43]. The insects are commonly found in terrestrial regions of the world, their larvae are widely used as protein feeds to animals due to the presence of proteins and lipids they contain. The worm has a varying life cycle ranging from 280 to 630 days. The larvae hatch after 10–12 days at 18–20 °C and mature after 3–4 months; this stage can last up to 18 months. The mature larva is a light yellow-brown in color with a length of 20–32 mm and weighs between 130 and 160 mg [44]. Mealworm insects have mostly been used in industries to manufacture feeds for pets and zoo animals, and sometimes as feed for other livestock. The feed from this insect is said to be nutritious in protein (40–60% dry weight), complete in amino acids, including essential amino acids such as lysine (5.2–6.8 g/100 g protein), methionine (1.6–2.5 g/100 g protein), fats (25–35% dry weight), unsaturated fats like omega-3 (α-linolenic acid) and omega-6 (linoleic acid), have low levels of cholesterol, have vitamins and minerals, be highly digestible, and confer a functional ability to the animals [45,46], in addition to also being rich in a variety of organic materials. Studies on livestock have indicated that the feeding of mealworms recorded an improvement in weight in fish and poultry [31,47,48].

Housefly larvae (*Musca domestica*) belong to the order Diptera and family Muscidae. These are found all over the world and feed on manure and organic waste. The house fly maggot has been studied as an alternative feed for domestic animals, mostly for poultry and fish [35,49]. Housefly maggot production thrives well in warm temperatures of (>25 °C) and moisture. The eggs hatch into larvae after 8–12 h and the larval stage lasts for about 5 days, while the pupal stage lasts for 4–5 days. Adult females can lay up to 500 to 600 eggs, and even more, and they can do so under controlled management [50]. The housefly maggot has been commonly used as fish feed in ponds.

Crickets (*Gryllidae*) are insects that belong to the order Orthoptera and family Gryllidae. They are generally edible and are consumed globally, especially in Africa, South America and Asia, and are sold in open-air markets and restaurants [51]. Crickets feed on organic materials and do well at 28–30 °C. Approximately 2000 insects can be raised in 1 m^2^. Crickets are normally collected in the wild, especially at night using artificial lighting or even in the morning when insects are still dormant due to low temperatures. Grasshopper (*Sphenarium purpurascens*) is an insect that belongs to the Pyrgomorphidae family and is often available across the seasons [52], and it is normally found in regions with temperatures ranging from 5 to 25 °C and altitudes higher than 2000 m above the mean sea level. Grasshoppers are regarded as an insect pest, which affects crops. They have a triangular head, a cephalic fastigium symmetrically divided by a median line, a robust and fusiform body, and a convex and saddle-shaped pronotum. The females are large with larger heads, shorter antennas and smaller eyes. They have various colors including green, black, grey or brown [53]. The desert locust (*Schistocerca gregaria*) is an edible insect that shows particular promise for arid region production, demonstrating very low land use requirements and low energy needs due to its natural adaptation to harsh environments [54]. They are generally brown or grey in color and move in a swarm. While current data show moderate GHG emissions compared to other insects, its ability to consume a wide variety of plant biomass makes it an attractive option for sustainable protein production, particularly in Africa and the Middle East, where it is native [55].

**Table 2:** Environmental impact comparison between insect species commonly used for feed.

Common and Scientific Names	GHG Emissions	Land Use	Water Use	Energy Use	Waste Conversion Efficiency	References
Black soldier fly (*Hermetia illucens*)	Low	Low	Very low	Moderate to high	Excellent	[56,57]
Mealworms (*Tenebrio molitor*)	Low	Low	Low	Moderate	Moderate	[33]
Crickets (*Acheta domesticus*)	Low	Low	Moderate	Moderate	Good	[14,58]
Grasshoppers (*Schistocerca gregaria*)	Moderate	Very low	Moderate	Low	Good	[59]
Housefly (*Musca domestica*)	Low	Very low	Very low	Moderate	Excellent	[60]
Desert locust (*Schistocerca gregaria*)	Moderate	Very low	Moderate	low	Good	[54]

## 6. Legislation and Regulation of Insect-Based Feed in Some Countries

Insect production and consumption have become common in many countries; despite this, the use of insects as feed and food has some challenges, including microbiological concerns which may arise from congestion within the insect production area; this may cause the spread of bacteria, fungi, and viruses for instance. For example, the investigation conducted by Vandeweyer et al. [61] through the Food and Consumer Product Safety Authority in the Netherlands on mealworms and locusts found out that almost a half of the insects under study contained aerobic bacteria exceeding 6 log CFU/g, and Enterobacteriaceae of more than 3 log CFU/g. In addition, some of the chemicals, like heavy metals including lead (Pb), arsenic (As), mercury (Hg), and cadmium (Cd), can also accumulate in the body of the insect, thus affecting the health of the animals fed on these insects. Additionally, some of the insects have allergenic materials such as chitin, for example, the silkworm, locust and grasshoppers, which can affect human beings when not well handled during production. These facts highlight the need of conducting thorough evaluations of insect feeds prior to their use as feed or food [62].

These challenges call for proper and enforced regulatory and legislative rules on the safety of their use as feed and food. Unfortunately, most developing countries, which are known to favor insect farming, lack regulatory policies on the use of insects as food and feed; they mostly rely on guidelines set up by the World Health Organization (WHO), Food and Agricultural Organization (FAO) and food and feed safety legislation by various governmental departments with no specific guidelines on insect use [23,63]. This is not the case with most developed countries, as they have important country-specific regulatory rules and legislation governing the use of insects as feed and food [64]. For instance, the Canadian Food Inspection Agency (CFIA) under the Feed Act and Feed Regulations (FAFR) emphasizes that any new feed materials in Canada must be certified by the authority before these are used and permits the use of black soldier fly as poultry and aquaculture feed [65]. The European Union Food and Safety Authority (EFSA) also stresses that any new feed material used in Europe must be approved by the authority. The EFSA has said that BSF, house fly, yellow mealworm, lesser mealworm, house cricket, banded cricket and field cricket insects can be used as fish food, but they must only be made from materials that are approved according to EU rules [66].

The Federal Food and Drug Administration (FDA) & Association of American Feed Control Officials (AAFCO) in the United State under The Federal Food, Drug, and Cosmetic Act (FFDCA) require the approval of new feed products and authorize the use of BSF as feed in aquaculture [66]. There have been initiatives by regulatory bodies to encourage the creation of rules for the production and use of insects in animal feeds throughout Latin America, taking into account the region’s rich insect variety [67,68]. Insect breeding and processing for sale as food or feed is unregulated in the majority of Latin American nations. Brazil, Argentina (by Argentine Food Code (CAA) Chapter XXIII), Colombia (by The National Institute of Drug and Food Surveillance (INVIMA)), Chile, and Costa Rica are among the countries that have begun drafting regulations to ensure the biosecurity of insects and their products [69].

In Korea, The Ministry of Agriculture, Food, and Rural Affairs (MAFRA) has a regulation act on the control of livestock and fish, which has the mandate to approve any new feed material in the country [70]. The Ministry of Agriculture, Forestry and Fisheries in Japan under the Safety Assurance and Quality Improvement of Feeds Act authorizes the use of any new feed material used in the country [66]. The Australian Pesticides and Veterinary Medicine Authority (APVMA) certifies any new feed substance utilized in the country, dependent upon compliance with the Good Manufacturing Practice, Australian animal feed industry codes of practice, and standards for animal feed manufacture [71]. The Ministry of Agriculture and Rural Affairs in China also has regulations on the administrative measures for feed and feed additives, which authorizes any new feed materials in the country [65]. When we look at the importance of insect production in the food and feed industry, there is a need to regulate its entire production process for the sustainability and safety of the users through the development of regulatory standards geared specifically to the use of insects, especially by developing countries who lack these policies.

## 7. Climate Change Mitigation Mechanisms of Insect-Based Feed

Because of their rapid development and remarkable efficiency, insect farming for feed is a more sustainable alternative to traditional feed production [72]. When compared to animals, insects are far better at breaking down organic materials into protein, and thus farming insects can help alleviate the issue of food loss and waste. Above all else, compared to traditional livestock, rearing insects requires far less land, water, and energy [73]. For example, the use of BSFL meal as a protein source in rainbow trout diet reduces land and water requirements and greenhouse gas emissions compared to SBM or FM, according to Smetana et al. [32]. Nevertheless, it increases energy consumption and could lead to water contamination from power usage and nutrient runoff, as well as ammonia emissions, which intensify environmental problems including acidification and eutrophication [74], but a better nutrient management for BSFL, e.g., organic waste, can lessen these negative consequences.

Insects can obtain all the water they require from the food they eat, making them more resistant to dry conditions; thus, the majority of water used in insect farming goes towards maintenance. The increased protein yield per land is also noteworthy, and the significant decrease in the emissions of greenhouse gases is a crucial advantage of insect farming. The shorter life cycles of insects mean that there is less time to reproduce, making insect farming an economically viable option. The process is also relatively simple, easy as regards transportation, and requires nothing in the way of long-term training. The annual economic advantage of using insect meal instead of conventional feed sources was projected to be EUR 34 million according to a study in Uganda [75,76]. In addition, it is possible to raise insects all year round with limited resources since they have practical breeding systems [77].

In addition to production sustainability and feed efficiency (protein and fat value, and functional compounds), IBF has also potential in reducing methane emissions in ruminant farming when used as a protein source in cattle diets, as IBF tends to influence rumen microbial activity, leading to low methane (CH_4_) production by converting dietary crude protein (CP) into microbial crude protein (MCP). In addition, some insect-derived proteins may also alter rumen microbiota composition, favoring propionate-producing bacteria over methanogens. This shift could directly suppress methane emissions while improving feed energy retention [78,79]. Research also indicates that IBF can directly suppress ruminant methane production by inhibiting fatty acids. The high polyunsaturated fatty acids contained in insect meal affects the cellulolytic bacteria and protozoa, altering their membrane function by reducing hydrogen availability for methanogenesis [3]. Additionally, chitin from insects limits methanogens’ access to water while altering rumen microbial composition, further reducing methane synthesis [80].

## 8. Insect-Based Feed in Ruminant for Methane Emission Reduction

IBF is becoming a promising nutritional option for mitigating enteric CH_4_ emissions in ruminant livestock; this is mainly achieved through the following biochemical mechanisms: chitin-mediated suppression of methanogens, which disrupts cell membrane functioning and competition for essential substrates [80]; long-chain polyunsaturated fatty acids (PUFAs), which suppress the protozoa responsible for interspecies hydrogen transfer, thereby limiting the availability of methanogenesis substrate [3]; and improved nitrogen utilization, which limits fermentable substrates, thereby reducing ammonia production and subsequent methanogenic activity [81]. Studies have shown that insect-based feeds consistently reduce methane emissions by 12–30% per unit of feed consumed in cattle, sheep and goats. This reduction is dependent upon the type of insect used, the amount of insect meal included in the diet (best results at 5–15% of feed), and the processing methods used during manufacturing [58]. This section evaluates these mechanisms in the selected ruminant species below.

### 8.1. Cattle

The substitution of soybean meal with insect-based protein feed can significantly reduce enteric methane emissions in cattle through interconnected ruminal modifications. For instance, Ahmed et al. [78] conducted a study in which, instead of SBM, ruminally fistulated non-lactating Holstein cattle was given 25% supplement of commercially available insect powder produced from adult field crickets (*Gryllus bimaculatus*, protein content 56.2%) and silkworm pupae (*B. mori*, protein content 52.4%). These supplements somewhat increased pH, reduced CH_4_ production (18 and 16%, respectively), and had no effect on nutritional digestion. The methane-suppressing effects are well explained by Phesatcha et al. [82], who reported 18% and 16% reductions in CH_4_ production when supplementing Holstein cattle with 25% cricket (*Gryllus bimaculatus*) and silkworm pupae (*B. mori*) meals, respectively, with these decreases directly correlating with increases in rumen pH and unimpaired nutrient digestion. This is consistent with increased protein degradation in the rumen and suggests that this supplement may be useful for identifying nitrogen metabolism in ruminants, as increased microbial protein synthesis lowers the rumen ammonia nitrogen concentration. In addition to a decrease in protozoal populations, they discovered that total volatile fatty acid (VFA) and propionate (C3) both increased significantly, but acetate (C2) and the C2:C3 ratio decreased significantly (*p* < 0.05).

The experiment on dairy cows demonstrated that BSFL, with its high energy value of 38.4 MJ, could be a viable alternative fat source due to its efficiency. The results showed that the rumen pH decreased, there was an increase in amylolytic activity, the total microbial mass increased (*p* = 0.16) due to infusoria growth, and there was no negative effect on rumen metabolism. Additionally, there was a better breakdown of fiber to volatile fatty acids (*p* < 0.05) and an improvement in amylolytic activity (*p* < 0.05). Furthermore, it resulted in a higher milk fat content (*p* = 0.16), an improved fat-to-protein ratio, and a reduction in ammonia production in the rumen (*p* < 0.05) [83]. A similar positive result was also observed in digestibility and in vitro rumen fermentation in Holstein cows by Kahraman et al. [84] supplemented with defatted BSFL meal to replace 20% and 40% of the SBM. The consistency in methane mitigation across these studies stems from three mechanisms: (1) insect-derived chitin suppression of methanogen activity, (2) polyunsaturated fatty acid disruption of hydrogen-producing protozoa, and (3) enhanced nitrogen efficiency that limits fermentable substrates for methanogenesis.

### 8.2. Sheep

Based on in vitro research in sheep [85], cricket oil (*Acheta domesticus*) offers the most potential for soybean oil due to its ability to raise the concentration of the potentially health-promoting trans-11 18:1 without changing the concentration of trans-10 18:1. Supplementing with 20 g/kg DM of cricket oil promotes the growth or activity-specific bacteria that are responsible for hydration and oxidation of ruminal FA. Additionally, the oils have an inhibitory effect on de novo FA synthesis by microbiota, which is caused by changes in the abundance or function of ruminal bacteria. In another study [79], not-so-significant results were found; fattening lambs supplemented with 60 g/kg DM of water fly (*Notonecta* sp.) meal had the lowest generation of NH3-N at hours 12 and 24 (*p* = 0.032 and 0.021, respectively), although it was not statistically significant when compared to other feed sources. There were no changes in CH_4_ generation, but there was a positive nitrogen balance (*p* = 0.0002), the greatest fermentation rate (*p* = 0.0007), and a tendency for urinary nitrogen excretion to decrease (*p* = 0.100).

In Indian Mandya sheep, the control group had a daily methane emission of 23.6 g/day, while the Mandya sheep groups that received daily supplementation or biweekly supplementation of silkworm pupae oil had a lower daily methane emission of 23–25% (*p* = 0.01). Likewise, the supplemented groups exhibited significant reduced methane production (g/kg DMI) when contrasted with the control group (*p* < 0.01). While the supplementation had no effect on overall VFA or acetate production, it did cause a change in the fermentation pattern, with more propionate and a lower acetate-to-propionate ratio. The oil had a long-lasting effect on the numbers of ruminal protozoa, but it had no apparent impact on the archaeal community composition [86].

### 8.3. Goats

According to the results obtained from the goat experiments [87], the BSF had an extremely high dry matter content of 973.3 g/kg, a protein content of 407.4 g/kg, and a fat content of 327.0 g/kg. The saturated fatty acid (SFA) content of BSF was high at 56.10 g/100 g, with the highest concentration of C12:0 at 41.9 g/100 g. The total unsaturated fatty acid (UFA) content was 18.50 g/100 g. The authors discovered that microbes’ nutrient utilization rate was improved with 5% and 10% BSF supplements; however, 15% supplementation produced the best results for reduction in CH_4_ production. The presence of chitosan, which has antibacterial capabilities, inhibited CH_4_ formation, while the high C12:0 level inhibited gas production. In Qianbei Ma’s study [88], goats that were given a 10% heat-treated BSF supplement had the greatest amounts of C18:1n9c in the *longissimus thoracis* and *lumborum* muscles (*p* < 0.05), a significant increase in UFA, and a significant decrease in SFA. But this supplement lowers the meat’s quality, which is a downside. The formation of starch–lipid complexes during heat treatment has the potential to prevent the breakdown of UFAs in the rumen and enhance their absorption in the small intestine, leading to an increase in the amount of UFA in the muscles.

In the study by Astuti et al. [89], the effects of cricket meal as a milk replacer on pre-weaning goat kids and as a substitute to soybean meals in the diets for post-weaning goats’ kids were examined. The authors found out that there was no significant difference in the pre-weaning performance of goat kids fed on the milk replacer containing the cricket meal as these had the same average daily gain on final body weight when compared to kids fed with goat milk. Similarly, the post-weaning goats fed on the cricket meal (up to 30% of the concentrate) did not show any significant variation in rumen fermentation or growth performance when compared to the post-weaning goats fed on the control concentrate containing soybean meal. This study concluded that cricket meal is palatable and has no effect on the goat’s health; therefore, it can be added to goat feed.

Additionally, in a different study, the use of BSF and cricket meal as part of a milk replacer has also been reported to increase the preweaning average daily gain (ADG) by about 100–120 g/h/d in goats and sheep. When the feeds are used as creep feeding, they can improve the post-weaning average daily gain by more than 150 g/h/d in sheep and goats [90]. In addition, incorporating mealworm frass of up to 3% in the feed mixture was reported to improve growth, feed utilization, and milk productivity in dairy goats [91]. Despite the limited study on insect meal in goats, the available studies have shown promising results on growth productivity; hence, more research is needed to include this in good feeds.

## 9. Challenges and Future Direction

**Figure 3:**
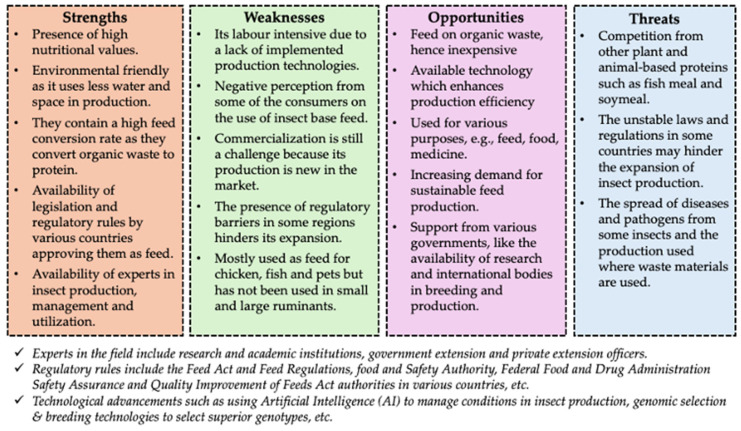
SWOT analysis of insect-based feed for sustainable livestock farming.

While there are many benefits to using insect-based feed in sustainable livestock production, there are also a few challenges (Figure 3). The main challenges in the implementation of IBF regard the level of acceptance of it, which might differ depending on the livestock value chain and the farmers’ circumstances. Odinya et al. [92] found that just 11% of Kenyan dairy farmers were familiar with IBF, suggesting that it is not currently well known or promoted within their sector. But when the option became available, many farmers were eager to utilize IBF; so, maybe awareness is more of a problem than outright rejection. A study in Ireland [93] found that while 81.7% of pig farmers and 71.8% of poultry farmers supported using IBF, just 53.5% of cattle farmers and 52.1% of sheep farmers were in favor, with safety and consumer acceptance being their primary concerns. Supporting that statement, Raccatello et al. [94] confirmed that consumers in the West still view insects as unorthodox, which limits their acceptability in diets. Even countries that adhere to religious restrictions for food (such as Halal in Islam) question food generated by IBF due to its association with impurity and filth [95].

Another challenge that could prevent insects from being widely used in animal feed is the unclear or absence of regulations regarding their use. Most developed countries, including the United States, the EU, Canada, China and Japan, completely outlawed the use of IBF as ruminant feed. However, the use of insects as a feed source for ruminants is not regulated in many countries, particularly in developing countries [15,78]. This ongoing debate around the use of insect-based feed for ruminants on a global scale is due to the concern that prion illnesses, such as spongiform encephalopathy, could be transmitted to animals. In particular, the EU forbids the use of animal manure as a base for raising insect larvae for the purpose of feeding domesticated animals [77].

Major reasons for the unclear regulations, probably due to safety-related standards, are still hard to determine since this area is relatively new and continually evolving. The nutritional composition of the final products can be affected by using different substrates for feeding insects, which makes it difficult to standardize these feeds. Maximizing their possible value as feed components depends on tailoring their diet throughout the larval period. The crucial point is also to employ locally generated, pre-consumer food waste as a substrate for insect rearing based on the idea that such waste has vital nutrients that would give a sustainable and nutrient-rich feeding option. Therefore, revealing insights that could greatly enhance their application in livestock nutrition depends on looking at several feeding substrates for insect farming and their consequent effect on the nutritional profile of the larvae. Consequently, better technologies and infrastructures need to be implemented to improve insect production for animal feed on farms. The goal is to achieve standardization of insect feeding, but these practices must be implemented appropriately to avoid negative environmental impacts [96,97,98].

## 10. Conclusions

The black soldier fly, mealworms, grasshoppers, houseflies, and desert locusts are among the many insect species that have been the subject of research on the reduction in methane emission in ruminants. Studies show the significant potential of various insect species in reducing livestock emissions by 50–90% as compared to conventional feeds, particularly when using waste products in production; their true value lies in being part of an integrated solution that simultaneously addresses climate change, resource efficiency, and food security challenges. The current review confirms that insect feed can directly lower greenhouse gas emissions through efficient production systems and potentially modulate rumen function in livestock, while other studies observed their ability to convert 30–60% of organic waste into high-quality protein. However, in order to realize the full importance of this insect-based protein feed, there is a need to have proper and enforced regulations that favor insects as a source of animal protein, the commercialization of insect production, and continued development of research on insect-based feed use that will make insects not just a future possibility but a solution for climate-resilient livestock production systems for the future.

## Data Availability

There are no data generated in this article.

## Abbreviations

The following abbreviations are used in this manuscript:

AAFCO	Association of American Feed Control Officials
ADG	Average daily gain
APVMA	The Australian Pesticides and Veterinary Medicine Authority
As	Arsenic
BSF	Black soldier fly
DMI	Dry matter intake
EUR	Euros
BSFL	Black soldier fly larvae
FA	Fatty acids
GHC	Green gas emission
BUN	Blood urea nitrogen
Ca	Calcium
Cd	Cadmium
CFIA	Canadian Food Inspection Agency
CP	Crude protein
Cu	Copper
EFSA	The European Union food and Safety Authority
FAFR	The Feed Act and Feed Regulations
FAO	Food and Agricultural Organization
FDA	The Federal Food and Drug Administration
FFDCA	The Federal Food, Drug, and Cosmetic Act
FM	Fishmeal
Hg	Mercury
IBF	Insect-based feed
K	Potassium
MAFRA	The Ministry of Agriculture, Food, and Rural Affairs
MCP	Microbial crude protein
Mg	Magnesium
Mn	Manganese
Na	Sodium
P	Phosphate
Pb	Lead
SBM	Soybean meal
SFA	Saturated fatty acid
UFA	Unsaturated fatty acid
VFA	Volatile fatty acid
WHO	World Health Organization
Zn	Zinc
